# Routine air sampling and culture in operating room for prevention of surgical site infection

**DOI:** 10.1017/ash.2025.99

**Published:** 2025-09-03

**Authors:** Seon Uk Choi, Seo Yeon Lee, Ji Yeoung Yim, Young Hwa Choi, Wee Gyo Lee

**Affiliations:** 1 Infection Control OfficeAjou University Hospital, Suwon, Korea; 2 Department of Infectious DiseasesAjou University School of Medicine, Suwon, Korea; 3 Department of Laboratory MedicineAjou University School of Medicine, Suwon, Korea

**Keywords:** Airborne bacterial count, Air sampling, Operating room

## Abstract

**Objective:** Open surgical wound is prone to surgical site infection due to contamination of surrounding environment. Therefore, routine air sampling and culture of two operating rooms (OR) was performed from 2018 to 2023 to monitor and evaluate air quality and provide appropriate infection control measures. **Method:** 2 OR regularly performing prosthetic insertion were selected for routine air sampling every 6 months due to high risk of surgical infection associated with the procedure. Air sampling was performed by collecting 1000 litre of air over 10 minutes using air sampler (MAS-100 Eco, Merck). Collected air was cultured on blood agar plate and Sabourand dextrose agar for 30 days, and pathogen identification and quantification was performed upon positive culture result. This study employed a cut-off point of 17.6 colony forming unit (CFU) as specified by federal standards on biological particles published by National Aeronautics and Space Administration. **Results:** 12 air samplings was performed from 2018 to 2023. A single case of positive bacterial air culture was reported (20 CFU, coagulase-negative *Staphylococcus*). Infection control measures were provided upon reporting of positive bacterial air culture, including inspection of positive pressure ventilation system and high efficiency particulate air filter, disinfection of OR and the equipment, and more strict regulation of temperature and humidity. Air sampling was repeated after imposing the measures to evaluate their effectiveness. Cases of surgical site infection caused by the identified pathogen were monitored for 90 days, after which it was determined that there was no surgical site infection related to positive air culture. **Conclusion:** The six-year monitoring of OR air sampling confirmed that detection of positive air culture in routine sampling was not associated with surgical site infection. Based on this result, the hospital decided to conduct air sampling and culture only in outbreak of surgical site infection as part of epidemiologic evaluation.

